# Avoidance Behaviours and Missed Opportunities in a Case of Metastatic Squamous Cell Carcinoma

**DOI:** 10.1155/2015/235943

**Published:** 2015-12-30

**Authors:** David Kelsey, Simon Zakeri, Chandra Hettiaratchi, Ndubisi Offonry

**Affiliations:** Barnet General Hospital, Royal Free NHS Foundation Trust, Barnet, Hertfordshire EN5 3DJ, UK

## Abstract

We describe the case of a 96-year-old woman who presented with a large fungating squamous cell carcinoma on her neck. In the Western hemisphere, it is rare to see patients with advanced tumours at their first presentation. We summarise the events leading to her late presentation to the hospital and explore the contributing factors. These may have included avoidance behaviour secondary to fears and misconceptions about cancer treatment. We conclude that healthcare professionals should be aware of these factors, and every effort should be made to address hidden fears and misconceptions when caring for patients with terminal illnesses. This will allay the patients' anxiety, thereby enabling them to make an informed choice about their future care.

## 1. Introduction

This case is an example of missed opportunities in cancer care in a hospital setting. It highlights the dangers of avoidance behaviours and poor communication. Avoidance behaviours are those characterised by actively ignoring a stressor in order to reduce mental discomfort [1]. Those at greatest risk of disease can be the least well informed and protected. This case demonstrates the potential harm of such behaviours and how they may lead to a late presentation to hospital. When coupled with poor communication and planning between healthcare professionals, conditions may become fulminant. This may lead to disastrous consequences for the patient.

In a society that often portrays cancer as a “battle,” there are those who have neither the will or the want to “fight.” Furthermore, in an information-rich world, there are still those who are completely dependent on others for information. With an ageing, often isolated population, more cases like this may slip through the net.

## 2. Case Presentation

The patient was a 96-year-old Caucasian woman who lived alone. She was visited by carers four times a day. She presented to our hospital two years after a recurrence of a right neck mass.

In 1999, she underwent a right partial mastectomy for ductal carcinoma (T2NXM0) and received adjuvant chemoradiotherapy. She remained disease-free and was discharged from follow-up ten years later.

In 2011, she was referred by her GP for a suspicious lesion on her right neck. This was diagnosed as a poorly differentiated squamous cell carcinoma and completely excised. She was discharged back to the care of her GP.

Between 2013 and 2015, she had been admitted on three occasions to a short-stay ward at a District General Hospital (Table 1). Of these, one was for presyncope and the others for urinary tract infections, anaemia, and general deterioration. She had been seen by geriatricians, general physicians, and rehabilitation staff. The patient declined investigation or intervention and was discharged with no follow-up.

**Figure 1:**
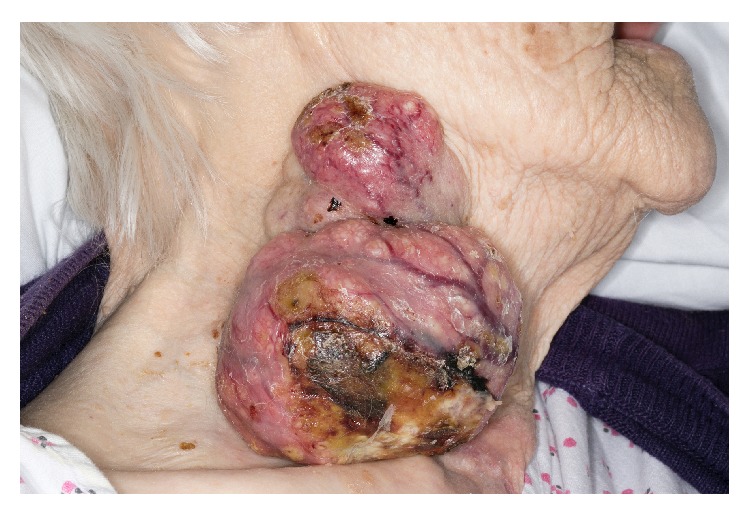
A lateral view of the mass.

In 2015, after being alerted to the patient's poor condition by her carers, the patient was visited at home by her GP. From there, the patient was referred to the Head and Neck MDT and to our hospital for admission. She presented to a long-stay care of the elderly ward with a bilobulated, fungating, 8 × 10 cm mass in the right neck (Figure 1). This mass had grown over a two-year period. Her principle complaints were of pain and the inability to lie comfortably.

She appeared cachectic, and apart from the neck mass physical examination was unremarkable. The patient was aware of the possible malignant nature of the neck mass. During the two years from its recurrence to her final admission, she had not sought medical attention for it.

She said she wanted to be “left alone,” fearing that surgery and anaesthetic would lead her to “die on the table.” She also expressed a general reluctance to be “interfered with.” When asked, she expressed a wish to be pain-free and to die at home. She was unaware of palliative approaches to treatment.

### 2.1. Investigations

Initial blood tests showed Hb of 80 g/L; MCV of 87.9 fL; CRP of 36 mg/L; and WCC of 8.4 × 109/L. Kidney function was deranged with a urea of 10.7 mmol/L and creatinine of 117 mmol/L. Following transfusion of two units of blood, her haemoglobin recovered to 126 g/L.

A plain chest radiograph showed a left upper lobe mass that was not present on radiographs two months previously. A CT chest/abdomen/pelvis demonstrated a 6 cm left upper lobe lung mass, a lobulated, likely nodal, 5 cm mass in the caecum, and the right neck mass which measured 8 cm × 10 cm.

A punch biopsy of her neck mass was arranged, which was reported as an invasive, poorly differentiated, squamous cell carcinoma.

### 2.2. Treatment

On admission, the patient was referred to the palliative care team. Pain control was rapidly attained with regular oramorph and subcutaneous morphine sulphate titrated against response.

Control of agitation was achieved with subcutaneous midazolam, and subcutaneous hyoscine butylbromide effectively reduced respiratory secretions.

The patient was reviewed by the Head and Neck MDT who recommended palliative radiotherapy, followed by fast-track referral to a nursing home. She developed a hospital acquired pneumonia and received two days of intravenous piperacillin-tazobactam as per hospital guideline. Spiritual support was provided by the hospital chaplaincy service which was funded internally.

### 2.3. Outcome

After input from the hospital chaplain, the patient appeared more settled and accepting of the inevitability of the outcome.

However, the decision to commence radiotherapy was subjected to two multidisciplinary team reviews. This had the effect of delaying the onset of this definitive palliative treatment.

As a consequence, the patient died before radiotherapy could be initiated. The final agonal event was a hospital acquired pneumonia ten days after admission.

## 3. Discussion

Although ultimately the patient received palliative input, this only occurred after a hospital admission for severe pain. This was caused by a breakdown in communication between healthcare staff, as well as a failure to adequately empower and educate the patient during previous admissions.

The patient's neck mass recurred two years prior to her death. During that time, she had three admissions to short-stay wards at a District General Hospital. There was no evidence of exploration of the patient's wishes or fears regarding treatment during these admissions.

Furthermore, there was no planned follow-up or evidence of communication with her GP. Though she had full mental capacity to decide treatment options on all admissions, the patient did not die in the place or manner of her choosing. She was not aware of palliative approaches available to her. Doctors were only aware of what she did not want, not what she did. More could have been done to address her hidden fears and misconceptions.

The patient's reluctance to seek help and her misconceptions of cancer therapy are examples of an avoidance coping strategy. Avoidance coping strategies are maladaptive responses to a stressor [1]. They are linked to anxiety caused by perceived inefficacies of therapy and powerlessness with regard to outcomes [2–5]. Avoidance coping leads to delayed presentations to healthcare professionals [6]. Negatively perceived past experiences of healthcare play a significant role in the development of such coping strategies. It is likely that the patient exhibited such behaviours as a result of her past experiences with breast cancer [7, 8]. Awareness of avoidance behaviours during previous admissions may have enabled doctors to appropriately tailor care to the patient's wishes.

The patient wanted to die pain-free in her own home. No advanced care plan was put in place to enable this. Advanced care plans are designed to provide appropriate care goals for an individual expected to deteriorate to an extent whereby capacity or communication is lost. These require discussion over time between the patient and their care providers [9].

Advanced care plans have been shown to improve end of life care and reduce psychological discomfort for the patient and their relatives [10]. Better communication between primary and secondary care providers may have enabled such a care plan to be put in place.

Though we must respect the autonomy of those with full mental capacity, we also have a duty of care to fully inform our patients. If physicians were more aware of avoidance coping strategies, they may be better placed to explore seemingly unwise decisions. Society at large also has a duty of care. In an information-rich society, our elderly population are often computer illiterate and so rely on what is verbally communicated to them. Media portrayals of cancer are often pugilistic, rarely mentioning death. Moreover, they rarely feature the elderly who have the highest incidence rates [11].

The patient died during a hospital admission that could have been avoided. Lack of understanding of avoidance behaviours resulted in an ineffectual exploration of the patient's wishes. Doctors and society at large could have done more to educate the patient.

## Figures and Tables

**Table 1:** A timeline of events.

1999	Received wide local excision and adjuvant chemoradiotherapy for breast cancer

2009	Discharged from oncology follow-up, disease-free

2012	Discharged from dermatology follow-up

Sep. 2013	Admitted for beta-blocker related syncope. No mention of the right neck lesion in discharge summaries

Sep. 2014	Admitted to a short-stay ward following a fall Treated for a urinary tract infection and anaemia The patient refused investigation of the right neck mass and is deemed to have full capacity

Jan. the 2nd of 2015	GP referred the patient to the Head and Neck MDT after being alerted to her general deterioration by her carers

Jan. the 7th of 2015	Referred to hospital for general deterioration and pain. CT-chest/abdomen/pelvis performed Referral to palliative care made

Jan. the 13th of 2015	Initial Head and Neck MDt discussion. Biopsy reported poorly invasive squamous cell carcinoma

Jan. the 20th of 2015	The Head and Neck MDT opt for palliative radiotherapy. The patient begins treatment for pneumonia

Jan. the 23rd of 2015	The patient died on the ward
